# Correction: Validation of depression, anxiety, and stress scales (DASS-21) among Thai nursing students in an online learning environment during the COVID-19 outbreak: A multi-center study

**DOI:** 10.1371/journal.pone.0327057

**Published:** 2025-06-25

**Authors:** Yuwadee Wittayapun, Ueamporn Summart, Panicha Polpanadham, Thanyaporn Direksunthorn, Raweewan Paokanha, Naruk Judabood, Muhamad Zulfatul A’la

In the Ethics considerations statement, the ethical approval reference number from the Walailak University Institutional Review Board is listed incorrectly. The correct ethical approval reference number is: WUEC-22-077-01.
