# Supplementary material for: Validation of depression, anxiety, and stress scales (DASS-21) among Thai nursing students in an online learning environment during the COVID-19 outbreak: A multi-center study
Source: PLoS One. 2023 Jun 30;18(6):e0288041. doi: 10.1371/journal.pone.0288041 (PMC10312998; doi:10.1371/journal.pone.0288041)
Supplement: Attachment — Submitted filename: Response to Reviewers.docx [file pone.0288041.s002.docx]

**Responses to reviewers on MS version dated 21 April 2023**

Validation of depression, anxiety, and stress scales (DASS-21) among Thai nursing students in an online learning environment during the COVID-19 outbreak: a multi-center study.

| ***Editor comments:*** | ***Response from author*** |
| --- | --- |
| 1-Please ensure that your manuscript meets PLOS ONE's style requirements, including those for file naming | ***Response 1:***  We have reviewed all our MS followed PLOS ONE's style requirements.  Please see the revised manuscript. |
| 2- During your revisions, please note that a simple title correction is required | ***Response 2:***  We removed the text "Ref. KPB.B 1900/225/2565 11 December, 2022" from the title on the online submission information.  Please see the revised manuscript. |
| 3- Please provide additional details regarding participant consent. In the ethics statement in the Methods and online submission information, please ensure that you have specified what type you obtained. | ***Response 3:***  We have described more information of participant consent in part of study design and population (Method). Since we collected data via the online survey, nursing students were able to receive participant information sheets online from us. By choosing the online option that said, "agree to participate in the research project,"  Please see the revised manuscript.  (Line 92-94) |
| 4- We note that you have provided funding information that is not currently declared in your Funding Statement. However, funding information should not appear in the Acknowledgments section or other areas of your manuscript. We will only publish funding information present in the Funding Statement section of the online submission form. | ***Response 4:***  We have already removed funding information that appeared in the Acknowledgments and Funding section and any section of our manuscript.  “The funders had no role in study design, data collection and analysis, decision to publish, or preparation of the manuscript.”  We will include our amended statements within our cover letter; we will change the online submission form on your behalf.  Please see the revised manuscript. |
| 5- In your Data Availability statement, you have not specified where the minimal data set underlying the results described in your manuscript can be found. PLOS defines a study's minimal data set as the underlying data used to reach the conclusions drawn in the manuscript and any additional data required to replicate the reported study findings in their entirety. All PLOS journals require that the minimal data set be made fully available. | ***Response 5:***  We have already generated our minimal data set 2,000 datasets out of the total (3,705). (D1.XLSX)  Please see the revised manuscript. |
| 6-We note that you have indicated that data from this study is available upon request. PLOS only allows data to be available upon request if there are legal or ethical restrictions on sharing data publicly | ***Response 6:***  After we reviewed about your data sharing policy, we agree to send our minimal data set according for your policy. |
| 7-While revising your submission, please upload your figure files to the Preflight Analysis and Conversion Engine (PACE) digital diagnostic tool, https://pacev2.apexcovantage.com/. PACE helps ensure that figures meet PLOS requirements. To use PACE, you must first register as a user. Registration is free. Then, login and navigate to the UPLOAD tab, where you will find detailed instructions on how to use the tool. If you encounter any issues or have any questions when using PACE, please email PLOS at figures@plos.org. Please note that Supporting Information files do not need this step. | ***Response 7:***  We formatted our figures following your suggestions.  Please see the revised manuscript. |

| ***Reviewer’s comments:*** | ***Response from author*** |
| --- | --- |
| 1- Is the manuscript technically sound, and do the data support the conclusions?  The manuscript must describe a technically sound piece of scientific research with data that supports the conclusions. Experiments must have been conducted rigorously, with appropriate controls, replication, and sample sizes. The conclusions must be drawn appropriately based on the data presented. | ***Response 1:***  After we reviewed all parts of our manuscripts followed the reviewer’s suggestion, we found some error of our manuscript and attempt to edited it in all parts of the reviewer suggestion such as  Sampling technique, the sample size calculation how to interpret the tool. We confirmed that the conclusions of our manuscript were drawn appropriately based on our data presented.  Please see the revised manuscript. |
| 2- Has the statistical analysis been performed appropriately and rigorously? | ***Response 1:***  We have been revised our statistical method followed your suggestion in the part of convergent validity by using AVE calculation and evidence of validity by CR and removed the VAS-F form the convergent validity because it measures in different construct of DASS.  Please see the revised manuscript. We will describe the additional details in specific comments. |
| 3- Have the authors made all data underlying the findings in their manuscript fully available? | ***Response 3:***  After we read your data sharing policy, we agreed to generate our minimal data with all the variables set that support our findings. (D1.XLSX)  Please see the revised manuscript. |
| 4.-Is the manuscript presented in an intelligible fashion and written in standard English?  PLOS ONE does not copyedit accepted manuscripts, so the language in submitted articles must be clear, correct, and unambiguous. Any typographical or grammatical errors should be corrected at revision, so please note any specific errors here. | ***Response 4:*** The revised MS has been copyedited by a university colleague with a strong scientific background in the biomedical and behavioral sciences, who is a native speaker with over 25 years' experience editing Thai research papers.  Please see the revised manuscript. |
| ***Reviewers' comments: Reviewer's Responses to Questions*** | ***Response from author*** |
| ***Comments to the Author*** |  |
| 1.-Under study design & Population: It is not clear how they recruit the participants in their study since the sample was taken from a larger multi-center survey, and how they randomize them. | ***Response 1:***  The revised MS has been obtaining the data from a one part of the larger multi-center study, that aimed aiming to assess the effect effects of online learning on the prevalence and factors associated with musculoskeletal disorders among Thai, Indonesian, Vietnamese, and Laos faculty members and students during the COVID-19 outbreak. We add more details about how to randomize participants into our study using multi-stage simple random sampling. (In part of study designs and population)  Please see the revised manuscript.  (Line 85-95) |
| 2-Do the survey was part of the larger study, or after the larger study completed, they recruit the sample and send them the survey of DASS-21 | ***Response 2:***  This study was part of this large project that finished to collect data. We recruit some samples and send them the survey of DASS-21.  Please see the revised manuscript.  (Line 85-86) |
| 3- To interpret these results is comparable to the original version of DASS-41 that calculated from the total scores for each sub-scale multiplying by two". This statement is not clear. & why have they used the 21 items scale since the original was 41 items? | ***Response 3:***  We described “Because the DASS-21 is a shortened version of the DASS (42 items), the final score of each scale was multiplied by two before being compared with the original DASS scale” The reason why we used DASS-21 because it is a short form, and the psychiatric properties is compatible for the original version for the usefulness of the respondents.  Please see the revised manuscript.  (Line 105-107) |
| 4- Reference number 24 is retracted. | ***Response 4:***  We fulfilled this retracted reference 24.  Suresh K, Chandrashekara S. Sample size estimation and power analysis for clinical research studies. Journal of human reproductive sciences. 2012;5(7):7-13. https:// doi.org/ 10.4103/0974-1208.97779. PMID: 22870008  Please see the revised manuscript.  (Line 462-464) |
| 5- The exclusion criteria are not clear in the manuscript. | ***Response 5:***  We add more details of exclusion criteria “Having inappropriate responses to items, e.g., responding to only a part of general information in the Thai Version of DASS-21 served as the exclusion criteria.”  Please see the revised manuscript.  (Line 127-129) |
| 6- Fatigue (VAS-F) is a different construct than anxiety and depression. So how it was used to test the convergent validity of the DASS-18. | ***Response 6:***  After we received your suggestion, we reviewed our convergent validity and removed this tool out of the analysis for convergent validity.  Please see the revised manuscript. |
| 7- It is not clear how randomization of the participants was done? | ***Response 7:***  We described how randomization the participants into our analysis in the part of sample and study design.  Please see the revised manuscript.  (Line 85-95) |
| 8- was mentioned that the parallel analysis revealed a single factor, then a 3-factor solution was created. how was this run? | ***Response 8:***  Thanks for your suggestion. However, we revised these error words in our manuscript.  “After randomizing the 2,000 participants for EFA, firstly, parallel analysis of the matrix indicated that a three-factor solution could be extracted.”  Please see the revised manuscript.  (Line 184-185) |
| 9- What was the factor loading for the deleted items and in which factors they are loading (the rule is to retain items if they had a factor loading of 0.4 or more). | ***Response 9:***  We added the criteria of factor loading.  “Factor loadings less than 0.5 were suppressed, and item cross loadings more than 0.2 were removed one at a time.”  Please see the revised manuscript.  (Line 140-141) |
| 10- Did the total variance changed after you remove the three factors? | ***Response 10:***  Yes, the total variance changes from 69.31% (DASS-21) to 71.31% (DASS-18).  “To ascertain the factorial structure of DASS-21 and the underlying dimensions comprising its 21 items. The initial analysis revealed a three-factor structure that explained 69.31% of the original data's variance.”  Please see the revised manuscript.  (Line 188-189) |
| 11- The title of Table 2 needs to be changed to "The title of the table should be Exploratory Factor Analysis of the DASS-18" | ***Response 11:***  We have changed the title of table 2 following your suggestion.  “Table 2. Exploratory Factor Analysis of the DASS-18”  Please see the revised manuscript.  (Line 198) |
| 12- in page 11 under the tittle Confirmatory factor analysis, the term "subscales" is used instead of "Items".. there is a big difference between both. | ***Response 12:***  Please accept our apologies, however, we edited all our error terms regarding your comments.  “Three items from the stress scale were eliminated (the remaining 18 items of DASS, thus DASS-18).”  Please see the revised manuscript.  (Line 202-203) |
| 13- What was Composite Reliability (CR) of the three factors as evidence of reliability | ***Response 12:***  We have calculated the CR of the three factors of DASS-18.  “The CR of the three domains of DASS-18 ranging from 0.830 to 0.935 indicated evidence of acceptable reliability.”  We add the CR of the three subscales of DASS-18 in Table 4 (Table 4. Reliability analysis of the DASS-18) (Line 214)  Please see the revised manuscript.  (Line 287-239) |
| 14. What was the Average Variance Extracted (AVE) for the scales? (this is considered a measure of convergent validity) | ***Response 14:***  We investigated the convergent validity of DASS-18 using the AVE. To indicate convergent validity, the AVE must be equal to or greater than 0.50, indicating that the construct's variance accounts for more than 50% of its variation. (Line157-159)  AVE calculations showed all the DASS-18 subscales achieved a convergence effect (with the AVE of depression = 0.504; the AVE of anxiety = 0.674 and the AVE of stress = 0.551). (Line219-220)  Please see the revised manuscript. |
| 15. What is the relation of gender to convergent validity? Gender is not a scale. how you consider is evidence of convergent validity? | ***Response 15:***  Thanks for your suggestion. After we reviewed our MS and found this error, we changed our analysis into the association between DAS-18 and demographic characteristic.  Please see the revised manuscript. |
| 16.- Table 3 has a lot of errors & empty cells. besides, this is a correlation table not a Convergent and discriminant validity for the subscale of the DASS-18. | ***Response 16:***  We revised this table and removed a Convergent and discriminant validity for the subscale of the DASS-18. We edited all the empty cells and error cells in this table. “Table 3. Association of the DASS-18 scores with demographic characteristics of the Thai nursing student sample” (Line232-233)  “Association of the DASS-18 scores among demographic characteristic and VAS-F” (Line222-226)  Please see the revised manuscript. |
| 17.- How did you measure the variable "Online Learning". it was mentioned in table 3. | ***Response 17:***  As shown in Table 1 the variable “online learning” was code 0= some semester 1= 100% (complete) online learning.  Please see the revised manuscript. |
| 18.- some nonscientific terms are used such as "directly linked".. "Values were discovered to exist"." comparable discoveries" | ***Response 18:***  We have already revised and edited all nonscientific terms following your comment by the native speaker.  Please see the revised manuscript. |
| 19.- I am not sure why table 5 was reported. it is a correlation table. they have already run the factor analysis. | ***Response 19:***  This table aimed to present the intercorrelation matrix among DASS-18 sub scales and VAS-F. However. We have added additional results for this table.  Please see the revised manuscript. |
| 20. How the temporal stability of the tool was measure. " the term is mentioned in page 13 under "Discussion". | ***Response 20:***  The temporal stability was tested using Pearson correlations calculation.  Please see the revised manuscript.  (Line 162-163) |
| 21- There are some typing and grammatical errors in the manuscript. | ***Response 21:***  We have carefully revised and checked for some typing and grammatical errors in the manuscript. Moreover, we consulted an experienced native speaker to improve our manuscript.  Please see the revised manuscript. |
| 22- How did the authors know that female had more depression? We can’t know that by simple running correlation. You need to do a T-test | ***Response 21:***  The statistical analysis to compare the difference between continuous variables (total scores of DASS-18) and other variables such as sex including 3 statistic methods such as mean difference (from t-test and coefficient from regression analysis). For Pearson correlation, we code gender variable 0=male and 1 = female for the association with DAS-18. We found that r=0.103 (p<0.001) means the female participants were positively weakly correlation with DASS-18 comparing with male participants. |
| 23. The authors have mentioned in the discussion that "no effect on both the DASS-depression and DASS-anxiety scales as we found no significant cultural problems with those two scales and there was no concern with the EFA findings as demonstrated by the statistical results". I think this couldn’t attributed to cultural factors only. This depends on the responses of the participants | ***Response 23:***  Thanks for your suggestion, we agree with you and add this term into our manuscript.  Please see the revised manuscript.  “Due to the effect of cultural factors and the responses of the participants on how individuals apprehend comprehend items of the DASS-stress scale, they have exhibited no effect on both the DASS-depression and DASS-anxiety scales.”  Please see the revised manuscript.  (Line 310) |
| 24.- The authors have mentioned that "The Thai version's convergent validity is supported by favorable correlations with VAS-F dimensions; correlations in this direction were anticipated which were expected to measure the same construct" Actually they are measuring different constructs, so they came up with this conclusion? | ***Response 24:***  After we revised how to measure convergent validity and found the error of our manuscript, we calculated the AVE and used these values to confirm the convergent validity of DASS-18.  “The convergent validity of the DASS-18 was examined using the AVE calculation of all three subscales. The results revealed that all sub-scales’ AVE were greater than 0.50, corresponding to the convergent validity acceptance criteria. These findings were also compatible with the findings of a study that aimed to validate the DASS-21 among Vietnam students in an e-learning environment [7].    Please see the revised manuscript.  (Line 319-323) |
| 25. the authors have reported that there is evidence of concurrent validity but it is not clear how it was measured (278 anxiety and depression are used in the same tool, so it can't be considered a concurrent validity. | ***Response 25:***  We revised this point and other points in the discussion part following our findings that change related to the statistical analysis.  Please see the revised manuscript. |
|  |  |

Notes: Thanks for all of your comments. These comments are very important to me to improve my manuscript and the next manuscript. I am very appreciative to learn and review what your comments.
