# Supplementary material for: Validation of depression, anxiety, and stress scales (DASS-21) among Thai nursing students in an online learning environment during the COVID-19 outbreak: A multi-center study
Source: PLoS One. 2023 Jun 30;18(6):e0288041. doi: 10.1371/journal.pone.0288041 (PMC10312998; doi:10.1371/journal.pone.0288041)
Supplement: Attachment — Submitted filename: Response to Reviewers.docx [file pone.0288041.s003.docx]

**Responses to reviewers on MS version dated 12 June 2023**

Validation of depression, anxiety, and stress scales (DASS-21) among Thai nursing students in an online learning environment during the COVID-19 outbreak: a multi-center study.

|  | ***Journal Requirements*** | | ***Response from author*** | |
| --- | --- | --- | --- | --- |
|  | 1-Please review your reference list to ensure that it is complete and correct. If you have cited papers that have been retracted, please include the rationale for doing so in the manuscript text or remove these references and replace them with relevant current references. Any changes to the reference list should be mentioned in the rebuttal letter that accompanies your revised manuscript. If you need to cite a retracted article, indicate the article’s retracted status in the References list and also include a citation and full reference for the retraction notice | | ***Response 1:***  We have carefully reviewed and rechecked all references in our MS followed PLOS ONE's style, and all any change of our references were mentioned such as DOI or PMID were reported in the rebuttal letter.  List of editing references:  4. Aguilar MV, Torres G. Making Sense of Online Classes during Quarantine due to the COVID-19 Pandemic: Students’ Perceptions from a Philippine University. Asia Social Issues. 2021;14(4):248066. <https://so06.tci-thaijo.org/index.php/wjss>  18. Teo YC, Yusuf A, Alice Lim WP, Ghazali NB, Abd Rahman H, Lin N, et al. Validation of DASS-21 among nursing and midwifery students in Brunei. Journal of Public Health. 2019;27(3):387-91. <https://doi.org/10.1007/s10389-018-0947-z>  25. Shevlin M, Miles JN. Effects of sample size, model specification and factor loadings on the GFI in confirmatory factor analysis. Personality and Individual differences. 1998;25(1):85-90. <https://doi.org/10.1016/S0191-8869(98)00055-5>  26. Hu Lt, Bentler PM. Cutoff criteria for fit indexes in covariance structure analysis: conventional criteria versus new alternatives. Structural equation modeling: a multidisciplinary journal. 1999;6(1):1-55. <https://doi.org/10.1080/10705519909540118>  PMID: 36787513  27. Kaiser HF. An index of factorial simplicity. psychometrika. 1974;39(1):31-6. <https://doi.org/10.1007/BF02291575>  29. Coker A, Coker O, Sanni D. Psychometric properties of the 21-item depression anxiety stress scale (DASS-21). African Research Review. 2018;12(2):135-42. <http://dx.doi.org/10.4314/afrrev.v12i2.13>    30. Musa R, Maskat R. Psychometric properties of depression anxiety stress scale 21-item (DASS-21) malay version among a big sample population. Mediterranean Journal of Clinical Psychology. 2020;8(1). <https://doi.org/10.6092/2282-1619/mjcp-2269>  PMID: 16004657  32. Beumeler LFE, Waarsenburg E, Booij S, Scheurink A, Hoenders H. Evaluation of a lifestyle intervention program in primary care on physical and mental health and quality of life of cancer survivors: a pilot study. European Journal of Integrative Medicine. 2018; 23:12018; 23:1-5. <https://doi.org/10.1016/j.eujim.2018.08.007>  Please see the revised manuscript. | |
|  | 1. In the Methods section please include the informed consent statement to reflect whether "written or verbal" informed consent was obtained from all participants for inclusion in the study | | ***Response 1:***  We included the description of the informed consent of the participants before the survey began in part of Study design and population. (Line 101-103)  Before the survey began, a statement of consent was obtained from all participants by permitting only those who pressed "I consent" to go to the questionnaires. A total of 5136 participants indicated their willingness to join the study.  Please see the revised manuscript. | |
| ***Reviewer’s comments:*** | | ***Response from author*** | |  |
| Introduction: The introduction section could be written with more clarity and focus on the research gap. Some statements are too generic and require context and clarification of terms. I have detailed them below:  1. The reference to Lee et al (2019) was used to support the statement about low performance at work or school, disruptions in social life, and even suicide, but the reference is a study evaluating the Depression Anxiety Stress Scales, which is not directly relevant. It would be helpful to provide more specific and relevant references to support these claims. | | ***Response 1:***  After we reviewed the reference to Lee et al (2019), this reference is supported for the evaluation of anxiety and depression screening. We have already added more detail to our manuscript.  (Line 32-36)  Please see the revised manuscript. | |  |
| 2- The statement about "more than a third of nursing students" and almost 'one half of these students' would be unclear without the total number of nursing students in the context of the study. Clarifying the total number of nursing students would provide better context. | | ***Response 2:***  We have been clarified the total number of participants enrolled in this study.  (Line 38)  Please see the revised manuscript. | |  |
| 3- It would be beneficial to provide a clear definition of 'remote learning' or 'online learning,' whichever terms the authors choose, to establish the context. Additionally, the terms "high intentions," "extensive training and learning processes" need further clarification and explanation. | | ***Response 3:***  We clarified and added more detail for the term that your recommendation such as 'remote learning.' and the term of extensive training by clinical setting.  (Line 42-45)  Please see the revised manuscript. | |  |
| 4.- The article mentions a research article explaining the high risk of infection exposure and fear of transmitting diseases among students, but it is not clear how this relates to the study's context of 'remote learning'. The connection between remote learning and these factors needs to be clearly established... | | ***Response 4: “***the high risk of infection exposure and fear of transmitting diseases among students’ this statement was supported ‘The levels of anxiety and depression were higher among nursing students than among those students from other disciplines” However, we explain more reasons that supported effect of  “Remote learning” on anxiety and depression in the discussion part.  (Line 321-325)  Please see the revised manuscript. | |  |
| 5. The sentence in line 42-43 requires evidence to support the claim made. It would be beneficial to provide supporting references or data to strengthen this statement. | | ***Response 5:***  The revised MS has been provided reference to support this statement.  (Line 47)  Please see the revised manuscript. | |  |
| 6.- There should be a brief explanation of DASS, its psychometric properties, and the difference between DASS-21 and the original DASS scale. Justifying the use of DASS-21 in this study would enhance the manuscript. | | ***Response 6:***  The revised MS has been given a brief explanation of Dass-42 and Dass-21in the  study instruments to support why we justify using the DASS-21.  Please see the revised manuscript.  (Line 65-66) | |  |
| 7- It is important to define the young adult age group being referred to in the study. Clarifying the specific age range would eliminate ambiguity and improve the precision of the research. | | ***Response 7:***  The revised MS has been given definition of young adult age group.  Please see the revised manuscript.  (Line 81) | |  |
| 8-Study instruments: I was unsure how the process of translating the original version into Thai was conducted, as the reference was to a DASS Brunei version. It would be helpful to provide information on who conducted the translation, whether there was any back-translation, and the measures taken to ensure there was no loss of meaning in the translation and to ensure consistency and validity. Additionally, information on the reliability and validity of the selected instruments, including the VAS-F scale, should be provided. Was there any pilot testing of the translated version? | | ***Response 8:***  The revised MS has been given the details of the Thai version of DASS-21 and the VAS-F.  Please see the revised manuscript.  (Line 109-110; 125-126) | |  |
| 9- Sample size calculation: Information on the sample could have been better. Who were these nursing students? What kind of remote learning did they engage in, how many hours, and what courses were included? How were they recruited? It would also be helpful to clarify whether students with existing CMDs (Common Mental Disorders) were excluded or not.  Line 130 - (group 1, n= 2,000, and group 2, n= 1,706). The total should be 3,706. Please verify this. | | ***Response 9:***  The revised MS has been given more information of the samples of this study including the inclusion and exclusion criteria followed your recommendations.  Please see the revised manuscript.  (Line 136-140)  We have been verifying the total number of participants who enrolled in this study.  (Line 142) | |  |
| 10- Statistical analysis: Can you include the statistical software used? I wasn't clear which items were removed and how the study ended up using DASS-18. It was only explained later on under the subheading "Confirmatory factor analysis." It would be helpful to signpost the reader by mentioning that this would be discussed later on. | | ***Response 10:***  We have included the statistical software used.  Please see the revised manuscript.  (Line 146-147) | |  |
| 11- The results and discussion sections are generally well-written. However, the statement in Line 274-276 should elaborate more on cultural factors and draw on relevant literature to support the discussion. Additionally, in relation to this statement, I wasn't sure what was meant by "no significant cultural problems." It would be beneficial to provide further clarification or explanation to ensure understanding. | | ***Response 11:***  We will try to give more details for this statement.  Please see the revised manuscript.  (Line 287-292) | |  |
